# Tobacco Smoke Mediated Induction of Sinonasal Microbial Biofilms

**DOI:** 10.1371/journal.pone.0015700

**Published:** 2011-01-06

**Authors:** Natalia Goldstein-Daruech, Emily K. Cope, Ke-Qing Zhao, Katarina Vukovic, Jennifer M. Kofonow, Laurel Doghramji, Bernardo González, Alexander G. Chiu, David W. Kennedy, James N. Palmer, Jeffery G. Leid, James L. Kreindler, Noam A. Cohen

**Affiliations:** 1 Department of Otorhinolaryngology, Head and Neck Surgery, University of Pennsylvania, Philadelphia, Pennsylvania, United States of America; 2 PhD Program Medical Science, Pontificia Universidad Católica de Chile, Santiago, Chile; 3 Facultad de Ingeniería y Ciencia, Universidad Adolfo Ibáñez, Santiago, Chile; 4 Department of Biological Sciences, Northern Arizona University, Flagstaff, Arizona, United States of America; 5 Department of Otorhinolaryngology-Head and Neck Surgery, Eye & ENT Hospital, School of Shanghai Medicine, Fudan University, Shanghai, People's Republic of China; 6 Department of Otorhonolaryngology - Head and Neck Surgery, University Hospital Centre, Zagreb, Croatia; 7 Children's Hospital of Philadelphia and Division of Pulmonary Medicine, Department of Pediatrics, University of Pennsylvania School of Medicine, Philadelphia, Pennsylvania, United States of America; 8 Philadelphia Veterans Affairs Medical Center, Surgical Services, Philadelphia, Pennsylvania, United States of America; University of Liverpool, United Kingdom

## Abstract

Cigarette smokers and those exposed to second hand smoke are more susceptible to life threatening infection than non-smokers. While much is known about the devastating effect tobacco exposure has on the human body, less is known about the effect of tobacco smoke on the commensal and commonly found pathogenic bacteria of the human respiratory tract, or human respiratory tract microbiome. Chronic rhinosinusitis (CRS) is a common medical complaint, affecting 16% of the US population with an estimated aggregated cost of $6 billion annually. Epidemiologic studies demonstrate a correlation between tobacco smoke exposure and rhinosinusitis. Although a common cause of CRS has not been defined, bacterial presence within the nasal and paranasal sinuses is assumed to be contributory. Here we demonstrate that repetitive tobacco smoke exposure induces biofilm formation in a diverse set of bacteria isolated from the sinonasal cavities of patients with CRS. Additionally, bacteria isolated from patients with tobacco smoke exposure demonstrate robust *in vitro* biofilm formation when challenged with tobacco smoke compared to those isolated from smoke naïve patients. Lastly, bacteria from smoke exposed patients can revert to a non-biofilm phenotype when grown in the absence of tobacco smoke. These observations support the hypothesis that tobacco exposure induces sinonasal biofilm formation, thereby contributing to the conversion of a transient and medically treatable infection to a persistent and therapeutically recalcitrant condition.

## Introduction

Chronic rhinosinusitis (CRS) represents a spectrum of inflammatory and infectious processes concurrently affecting the nose and paranasal sinuses [1]. Recent reviews reported an estimated prevalence in the United States of 16% with an aggregated annual cost of nearly $6 billion [2], [3]. CRS patients may have significant decrements in quality of life, both in disease specific areas and in general health. In fact, patients requiring sinus surgery demonstrate worse scores for physical pain and social functioning than those suffering from chronic obstructive pulmonary disease, congestive heart failure, back pain, or angina [4].

A broad range of factors can contribute to the evolution of CRS symptoms. For example, inherent mucociliary defects such as those found in patients with cystic fibrosis or primary ciliary dyskinesia [5], [6], abnormal innate immunity [7], paranasal sinus anatomic variations [8], [9], environmental exposure [10], allergy, [11], [12] and microbial colonization [13], [14] have all been associated with CRS. Recently, multiple investigations have highlighted a possible role of bacterial biofilms in the persistence of chronic infections including CRS [15], [16], [17], [18], [19], [20].

Bacterial biofilms comprise a complex, organized community of bacteria that attach to both biotic and abiotic surfaces [21]. Biofilm-forming bacteria are thought to begin as independent, planktonic bacteria which become sessile and initiate biofilm formation by adhering to a surface and forming microcolonies. When a critical density of bacteria is reached, intra- and inter-bacterial species cross-talk begins through a process commonly referred to as quorum sensing, which comprises changes in gene expression and post-translational modification of proteins that ultimately lead to expression of the biofilm phenotype [22], [23]. This phenotype is characterized morphologically by the formation of microbial ‘towers’ that are composed of layers of embedded, live bacteria with intervening water channels, and a ‘mortar’ for these structures composed of bacterially produced exopolymeric matrix (carbohydrates, proteins and nucleic acids), making up as much as 90% of the biofilm volume.[24] Biofilms allow for the evasion of host defenses, decreased susceptibility to antibiotic therapy, and deliberate release of planktonic bacteria, which may result in implantation and colonization of new anatomic locations thereby causing nascent acute infections in the host [25].

In addition to genetic, anatomic, environmental and microbial contributions to the development of CRS, the specific environmental exposure to tobacco smoke has also been suggested as a risk factor for the “aggravation and prolongation of sinusitis” as far back as 1964 in the Surgeon General's report on smoking [26]. Several epidemiologic studies have demonstrated a higher prevalence of CRS in cigarette smokers as compared to non-smokers [27], [28]. Additionally, studies have evaluated the role of tobacco smoke exposure on outcomes of functional endoscopic sinus surgery (FESS) and demonstrated higher surgical revision rates and more frequent bouts of post surgical rhinosinusitis in smokers compared to non-smokers [29], [30], [31], [32]. While the detrimental effects of tobacco smoke exposure on the respiratory epithelium are well described [33], [34], [35], [36], the effects of tobacco smoke exposure on biofilm formation in bacteria from the paranasal sinuses has not been investigated.

Here, we report that repetitive *in vitro* exposure to whole tobacco smoke induces biofilm formation in bacteria isolated from the sinonasal cavities of patients with CRS. Additionally, bacteria isolated from patients with tobacco exposure demonstrate robust biofilm formation when challenged with tobacco smoke *in vitro* compared to bacteria isolated from smoke naïve patients. Lastly, bacteria from smoke exposed patients can revert to a non-biofilm phenotype when grown in the absence of tobacco smoke. The implication of our data is that smoke exposure *in vivo* induces alterations in the bacterial life cycle resulting in resistance to both host defenses as well as conventional antimicrobial therapy leading to a persistent infection that is much more difficult to treat. Furthermore, these findings have important ramifications not only for CRS, but for numerous other respiratory tract infection including otitis media, chronic obstructive pulmonary disease, bronchitis, and pneumonia.

## Materials and Methods

### Specimen collection

Approval was obtained from the University of Pennsylvania Institutional Review Board to enrol adult patients who met the objective and subjective guidelines for CRS, set forth by the Sinus and Allergy Health Partnership [37] and written consent was obtained from all patients participating in the study. Sinonasal cultures were obtained from CRS patients with mucopurulent sinonasal secretions. Participants in this study were solicited from patients undergoing evaluation for CRS at the University of Pennsylvania, Department of Otorhinolaryngology – Head and Neck Surgery. Subjects were consenting adults (both male and female) over the age of 18 years. Exclusion criteria included rhinologic granulomatous disease, cystic fibrosis, immune deficiencies or other genetic disorders that may directly affect mucociliary function such as primary ciliary dyskinesia. Patients were stratified based on past or present tobacco use, and whether or not each lived with a smoker. Duplicate sinonasal culture swabs were obtained under endoscopic guidance and performed either in the outpatient clinic setting, or during endoscopic sinus surgery. One bacterial swab was sent for microbiologic characterization and antibiotic sensitivities to the clinical microbiology laboratory and the other swab was placed in Luria-Bertani (LB) broth and grown overnight at 37°C. Following overnight growth, an additional bacterial swab of the resultant culture was obtained and sent for microbiologic characterization by the clinical microbiology laboratory by standard techniques. Commercially available strains *P. aeruginosa* (PAO1) and *Staphylococcus aureus* 29213 were purchased from ATCC.

### Biofilm quantification

Quantification of biofilm formation was performed as previously described [38]. Briefly, bacterial strains isolated from different patients, as well as the control strains, were grown overnight in 100% LB broth Luria- Bertani (LB) broth (Fisher Scientific, Hanover Park, Illinois, USA) at 37°C. The following morning cultures were diluted to an optical density of 600 nm (OD_600_)  = 0.1 and subsequently diluted (distilled water) 1∶100 in 50% LB broth, resulting in a final testing inoculum of ∼10^6^ CFU. One hundred fifty-µl of the freshly diluted culture was placed in octuplet in two flat-bottomed 96-well plates (Costar-Corning, Sigma-Aldrich Corp. St. Louis, MO, USA), one for tobacco exposure and one for sham exposure (see below). Biofilms were grown and quantified as previously reported [39], [40]. Briefly, following tobacco or sham exposure, bacteria were incubated in the 96-well plates for 20 h at 37°C. After incubation, the contents of each well were decanted and washed three times with 200 µl of sterile phosphate-buffered saline solution using a multichannel micropipette to remove all non-adherent bacteria but preserving the formed biofilm [38]. The remaining attached bacteria were heat-fixed by incubating at 60°C for 60 min. Subsequent quantification of biofilm was performed using the modified Christensen's method [41]. Each well of the 96 wells plate was filled with 150 µl 10% crystal violet (Harleco, Gibbstown, NJ), and incubated for 30 min at room temperature. The wells were then decanted and the excess dye was rinsed by placing the plate under running tap water until the water was clear. The dye bound to the adherent material was resolubilized and eluted with 150 µl of 95% ethanol per well at room temperature for 30 minutes, without shaking. Finally, the OD_595 nm_ of the ethanol elutions was determined using a BioRad 680 plate reader (Hercules, California). Each experiment was repeated a minimum of two times.

### *In vitro* smoke exposure

Freshly diluted cultures were placed with the lid off into an airtight box (20 cm (l) ×20 cm (w) ×15 cm (h)) with an inflow port at the top center, and a diffuser midway between the inflow port and the 96-well plate. Tobacco smoke was generated as previously described [35]. Briefly, standardized research cigarettes 1R5F (Tobacco and Health Research Institute, University of Kentucky) were ignited in an automated smoking machine (Teague TE-10, Teague Enterprises, Davis, CA), that was programmed to take a 2 s, 35 ml puff from the burning cigarette every 60 s. A total of five cigarettes were burned with the “inhaled” smoke being directly pumped into the exposure box. Bacteria were exposed to tobacco smoke for 3 h and then incubated in a designated 37°C “smoke” incubator. Sham exposed cells were placed in similar boxes (albeit never having been used for tobacco smoke exposure) with room air being vented in a similar fashion. After 3 h, sham-exposed plates were incubated in a separate incubator. Following 17 h of recovery, plates were processed as described above to score them for biofilm formation. Each experimental condition was performed a minimum of two times.

### *In vitro* chronic smoke exposure

Twenty hours after the initial exposure 50 µl of each strain from both the sham exposed and tobacco-exposed plates were removed and diluted 1∶100 in 50% LB broth. One hundred and fifty µl of the freshly diluted cultures were transferred to a new sterile flat-bottomed 96 wells plate for subsequent tobacco or sham exposure as detailed above. The procedure was repeated daily over the designated time course.

### Data Analysis

Data were expressed as mean ± standard deviation. Mann-Whitney U test and the chi-square test were done in order to analyze the relationship among variables and for the comparison of means and proportions. One way ANOVA, Kruskal-Wallis test and Dunn's Multiple Comparison Test were performed to analyze “phenotypic switch”. Differences were considered statistically significant when p<0.05.

## Results

### Biofilm formation in the absence or presence of exogenous tobacco smoke

To evaluate the degree to which tobacco smoke impacts biofilm formation, bacterial cultures were obtained from CRS patients, with and without tobacco exposure, demonstrating mucopurulent sinonasal secretions on nasal endoscopic exam. Culture swabs were obtained with endoscopic guidance and placed in appropriate media and grown overnight followed by microbial identification tests and biofilm formation assays. Taxonomical identity and source of the isolates is shown in Table 1. In addition to the clinical strains, commercially available strains of *Pseudomonas aeruginosa* (Pa) (PAO-1) and *Staphyloccocus aureus* (Sa) (29213) were used as positive controls for biofilm formation and to evaluate the effect of tobacco exposure on non-clinical isolates.

**Table 1:** Bacterial isolates used in this work.

Non-Smokers				Smokers			
ID	Biofilm Cx	Clinical Cx	PY	ID	Biofilm Cx	Clinical Cx	PY
1535	*S. aureus*	S. aureus*P. vulgaris*	None	1176	*K. oxytoca*	*K. oxytoca,* *C. freundii*	60
1580	*P. aeruginosa*	P. aeruginosa	None	1620	*S. pneumoniae*	*S. pneumoniae*	80
1662	*S. aureus*	*S. aureus*	None	1670	*P. vulgaris*	*P. vulgaris* *S. pneumoniae*	8
1750	*S. aureus*	*S. aureus*	None	1675	*S. aureus*	*S. aureus*	25
1751	*K. oxytoca*	*K. oxytoca* *S. liquifaciens*	None	1690	*S. aureus*	*S. aureus*	SHS
1752	*S. marcescens*	*S. marcescens*	None	1695	*S. aureus*	*S. aureus*	7
1754	*K. pneumoniae*	*K. pneumoniae P. vulgaris*	None	1696	*E. coli*	*E. coli*	12
1756	*S. pneumoniae*	*S. pneumoniae*	None	1843	*S. aureus*	*S. aureus*	10
1759	*S. aureus*	*S. aureus*	None	1848	*K. pneumoniae*	*K. pneumoniae*CNS	30
1760	*CNS*	*CNS*	None	1851	*S. pneumoniae*	*S. pneumoniae*CNS	18
1764	*CNS*	Enterococcus	None	1854	*P. aeruginosa*	*P. aeruginosa*	35
1772	*P. vulgaris*	*P. vulgaris*	None	1880	*K. pneumoniae*	*K. pneumoniae*	25
1775	*S. aureus*	*S. aureus*	None	1924	*P. aeruginosa*	*P. aeruginosa*	5
1779	*K. pneumoniae*	*K. pneumoniae* CNS	None	1951	*S. aureus*	*S. aureus*	12
1781	*S. marcescens*	CNS	None	2008	*S. aureus*	*S. aureus*	10
2001	*P. aeruginosa*	*P. aeruginosa*	None	2033	*S. aureus*	*S. aureus*	3

Culture result represents the bacterial isolate recovered from the initial Clinical culture or resultant from the Biofilm culture. Tobacco exposure is expressed a packs of cigarettes smoked per day X years smoking (PY). One patient, #1690, lived with a smoker and was thus designated as second hand smoker (SHS). CNS: Coagulase negative *Staph*.

**Figure 1:**
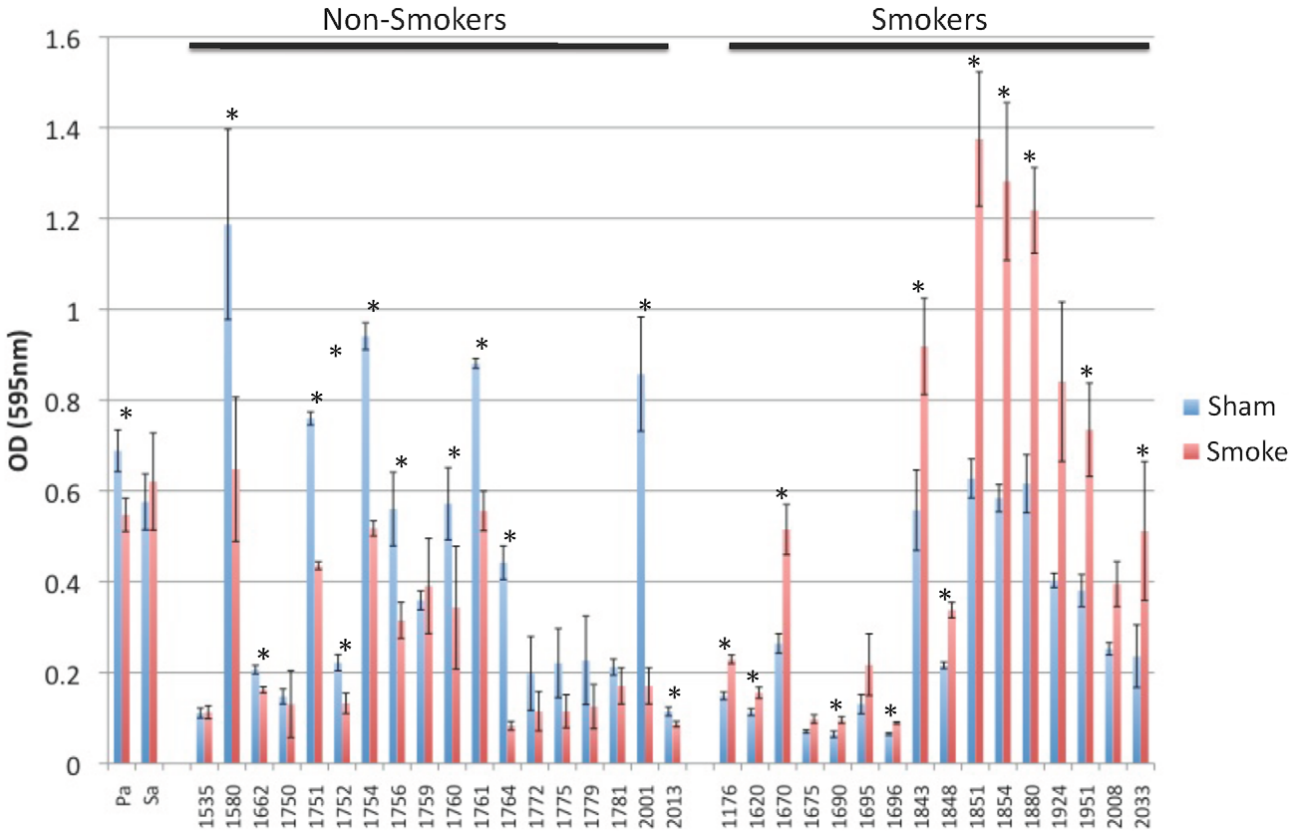
Biofilm formation in bacteria obtained from endoscopically guided sinonasal cultures, following sham or tobacco smoke exposure. Samples from patients evaluated in the outpatient clinic or in the operating room, who were found to have sinonasal mucopurulence were cultured. Samples were grown overnight and subjected to evaluation for biofilm forming capacity. A three hour tobacco smoke exposure (resultant from five cigarettes). Each isolate was performed in octuplet. A paired Student t-test was applied to compare smoke and sham exposed (* = p<0.01).

**Figure 2:**
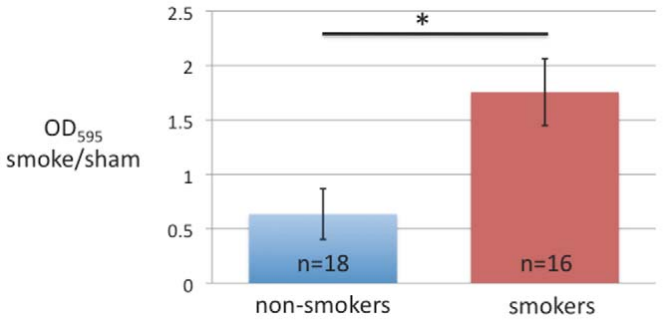
Tobacco biofilm index. Data from figure 1A was normalized by creating a ratio of smoke to sham exposed biofilm formation. Value of <1 demonstrates biofilm inhibition while value >1 reflects biofilm induction.

To determine whether the bacterial biofilm formation was altered by acute exposure to tobacco smoke, bacteria from either smoke-exposed patients or smoke-naïve patients were either sham or tobacco smoke challenged for three hours before undergoing the biofilm formation assay. Figure 1 demonstrates that acute smoke exposure significantly increased biofilm formation in 12 of 16 clinical isolates from smokers, but 0 of 18 isolates from non-smokers (* p<0.05). Because each isolate served as its own control, the ratio of biofilm formation (OD_595_ smoke/sham) represents induction of biofilm formation when greater than 1 or inhibition of biofilm formation when less than 1. *Ex vivo* smoke exposure of bacteria obtained from smokers' sinuses resulted in significantly more induction of biofilm formation (ratio of 1.75±0.31) than that seen in bacteria from nonsmokers' sinuses, in which *ex vivo* smoke exposure inhibited biofilm formation (ratio of 0.63±0.23) (figure 2, p<0.001). To determine whether the *in vivo* quantity of tobacco smoke exposure impacted on *in vitro* biofilm formation we evaluated tobacco exposure, as self reported by the patients in pack year history, to biofilm formation which revealed no correlation (r^2^ = 0.098) (data not shown).

### Biofilm formation after repetitive smoke exposure

**Figure 3:**
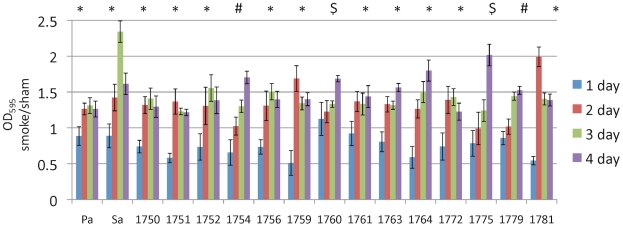
Biofilm formation after repetitive smoke exposure. Bacteria isolated from smoke naïve patients were subjected to daily sham or smoke exposure (5 cigarettes/3 h) and then subjected to the biofilm detection assay. Data is represented as the ratio of smoke exposure to sham exposure. *, #, and $ indicate significant differences (p<0.05) between day 1 and 2, day 1 and 3, and day 1 and 4, respectively. Indicate the number of replicates.

Because a single smoke exposure resulted in opposite effects on biofilm formation in bacteria isolated from smoke naïve patients vs. smoke-exposed patients, we hypothesized that chronic or repetitive smoke exposure would alter the biofilm forming capacity of bacteria. To test this hypothesis, we exposed bacterial cultures obtained from smoke naïve patients to smoke or sham daily (5 cigarettes over 3 h), for 4 days. Biofilm formation was tested 17 hours following each exposure. In support of our hypothesis, all 14 of the clinical isolates tested had a biofilm formation ratio of less than 1 after the first smoke exposure, but had a ratio greater than 1 by the fourth exposure (figure 3). In 10 of 14 clinical bacterial isolates and the two commercial controls (*P. aeruginosa* and *S. aureus*) showed a fast shift from tobacco induced biofilm inhibition (values <1) to tobacco induced biofilm formation (values >1) following the second day of smoke exposure (*) whereas the other three bacterial cultures required three (#) or four days of smoke exposure ($) (figure 3).

**Figure 4:**
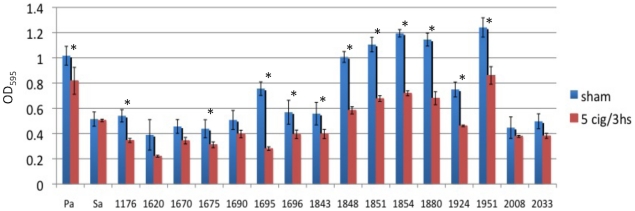
Smoke induced biofilm formation is reversible. Bacteria isolated from smokers were grown for 4 days in the absence of tobacco smoke, before a single sham or smoke exposure (5 cigarettes/3 h) and subsequent biofilm detection assay. Indicate the number of replicates, and repeat the significance test performed, and the p values.

The converse of our hypothesis is that bacteria from smokers' sinuses when removed from the stimulus of cigarette smoke revert to a phenotype similar to that seen in bacteria from nonsmokers. To test this possibility, we grew bacterial isolates from smokers in the absence of tobacco smoke for 4 days. On the fifth day we exposed them to tobacco smoke (5 cigarettes/3 h) and assessed the effect on biofilm formation 17 h later, similar to the experiments demonstrated in figure 1. In contrast to the data presented in figure 1, 12 of 17 clinical isolates from smokers had significant cigarette smoke inhibition of biofilm formation after growth in the absence of smoke for several days (figure 4).

## Discussion

The World Health Organization estimates that tobacco use accounts for 3% of the world's morbidity and mortality at a cost of tens of billions of U.S. dollars annually [42]. Although tremendous strides have been made in curtailing cigarette consumption, the prevalence of smoking among adults and children in the US remains approximately 22-24%, translating to an estimated 66,000,000 people who smoke regularly [42]. While much is known about the adverse effects of tobacco exposure on human physiology, relatively less is known about the effect of tobacco smoke on the respiratory tract microbiome. Recent work has demonstrated that cigarette smokers and those exposed to second hand smoke are more susceptible to life-threatening infection than non-smokers [33] and that smoking is an independent risk factor for pneumococcal pneumonia, Legionnaire's disease, periodontal disease, and meningococcal disease, among others [33], [43], [44].

CRS is a disease of unknown etiology which affects more than 35 million Americans of all ages resulting in over 500,000 surgeries in the US alone [45]. Patients with CRS may have significant decrements in quality of life, both in disease specific areas and in general health. In fact, patients requiring sinus surgery demonstrate worse scores for physical pain and social functioning than those suffering from chronic obstructive pulmonary disease, congestive heart failure, back pain, or angina [4]. This disease is most likely not one disorder, but rather represents a number of discrete entities and pathologies. Regardless of the underlying etiology, multiple reports have correlated tobacco smoke exposure with increased prevalence of CRS and poor sinus surgery outcomes [27], [28], [46]. The contributory mechanisms of tobacco smoke exposure to this disease process have predominately focused on its detrimental effects on the respiratory epithelium [34], [35], [36], [47], [48] and immune system [33], [49], [50], [51], but relatively less is known about the effect of tobacco smoke on the bacteria that reside in the sinonasal cavity which are felt to contribute directly to the pathogenesis of CRS.

A primary function of the nasal cavity is to humidify and cleanse inspired air. This is accomplished by creating transitional air flow (partially laminar and partially turbulent) which promotes the deposition of particulate matter in the sinonasal mucus blanket [52]. Thus, the nose and paranasal sinuses (especially in the post surgical cavity) are exposed to significantly higher concentrations of particulate environmental pollutants than are the lower airways [53]. Recently, multiple investigations have highlighted a possible role of sinonasal mucosa bacterial biofilms in persistent recalcitrant CRS [15], [16], [17], [18], [19], [20]. Thus, the effect of tobacco smoke on the ability to form biofilms was assessed in bacterial cultures obtained from smokers and smoke naïve CRS patients.

Utilizing endoscopically guided sinonasal microbial swabs from CRS patients with and without tobacco smoke exposure we assembled a battery of 34 microbial cultures (18 smoke naïve, 16 smoke exposed) (Table 1), which are representative of the microbes isolated from patients with CRS [18], [19], [54]. We are aware that the subsequent culturing protocol, i.e., overnight growth in broth prior to the smoke exposure, most likely selected for specific microbes and thus may not be fully reflective of the *in vivo* microbial milieu. We did not see a difference between the non-smokers and smokers in the bacteria cultured from the initial sinonasal swab nor the resultant bacteria of the biofilm cultures with the exception of one *E.coli* isolate. In general there was strong concordance between the initial clinical swab and the resultant biofilm culture. In four samples from smokers and four samples from non-smokers, the initial clinical cultures yielded more than one organism but the resultant biofilm culture only yielded one organism. Of the samples with multiple organisms identified, the second organism that did not propagate to the biofilm was coagulase negative *staph* (n = 3), *P. vulgaris* (n = 2), *S. liquifaciens* (n = 1), *C. freundii* (n = 1), and *S. pneumoniae* (n = 1). While Brook noted more pathogens recovered in nasopharyngeal swabs of smokers compared to non-smokers [55], in our CRS population we did not see a difference between cohorts. Furthermore, while many experimental paradigms investigating the role of tobacco exposure on biological processes utilize either cigarette smoke condensate [56] or cigarette smoke extract [44], we utilized whole cigarette smoke, as previously described [35], to better represent microbial *in vivo* exposure.

Our results clearly demonstrate that immediately following removal, bacterial isolates from smokers were more prone to produce biofilm material in response to smoke exposure than those from non-smokers, but that the latter group strongly enhances its ability to produce biofilms when repetitively exposed to smoke *in vitro*. Importantly, growth of the bacterial isolates from smokers in the absence of tobacco smoke produced a biofilm formation phenotype characteristic of the bacterial isolates from non-smokers (figure 4), suggesting reversibility of the tobacco effect and further supporting the notion that encouraging people to stop smoking has immediate positive health effects. Moreover, these phenotypic switches fostered by tobacco smoke exposure or removal, were not identified in a single organism but rather in more than eight different species. Therefore, we speculate that these responses represent a well-conserved, global microbial response to tobacco smoke exposure and could possibly represent a novel therapeutic target.

In fact, recent evidence suggests that smoking uptake and cessation can alter microbial communities [43], [57]. Although the exact mechanism(s) have not been elucidated, studies have demonstrated that cigarette smoke alters gene expression in microbial pathogens [44], [50]. For example, Bagaitkar *et al* used a whole genome microarray of *Porphyromonas gingivalis* to demonstrate that exposure of bacteria to cigarette-smoke conditioned medium caused differential expression of 6.8% of the *P. gingivalis* genome, including increase expression of virulence factors, alteration of expression of membrane proteins, and differential expression of oxidative stress genes [44].

Notably, evaluation of tobacco exposure, as self-reported by the patients in pack year history, did not correlate with the smoke induced biofilm formation (r^2^ = 0.098) (data not shown). This may be due to poor patient history, as studies have demonstrated that self-reported personal history is not always reliable as a means of screening for smoking [58], [59]. However, another possibility may be that smoke induced biofilm formation is triggered by a threshold exposure and is not a dose dependent phenomenon.

Our data was generated by whole tobacco smoke and thus is especially pertinent to the upper airways and possibly the lower airways, though certain caveats must be acknowledged. First, we chose empirically to expose bacteria to the smoke of 5 cigarettes over 3 hours with each cigarette being drawn by a 35-ml, 2-s puff once per min for 10 min. We chose this amount using the logic that a 1 pack per day smoker who smokes during 12 hours of the day will consume approximately 1.5 cigarettes per hour on average. This degree of exposure is most likely an exaggeration of *in vivo* sinonasal exposure, particularly as we did not ventilate the exposure chamber for three hours. Additionally, we did not vary the exposure time or dose to determine the threshold necessary for biofilm induction. Finally, we did not attempt to fractionate whole tobacco smoke and isolate the individual components responsible for microbial biofilm induction. However, if the biofilm induction activity is due to soluble factors of tobacco smoke, the ramifications of our findings far exceed respiratory infections, as the vascular system is well equipped to transport such soluble factors throughout the body. Future work will focus on identifying a threshold for tobacco induced biofilm formation as well as identifying the responsible component(s).

Taken together these data support the notion that tobacco smoke exposure induces biofilm formation in respiratory bacteria and that smoking cessation should revert bacteria back to a smoke naïve phenotype. *In vivo* this may translate into microbial community diversity. In support of this, studies have demonstrated that smoking cessation alters oral/subgingival microbial communities [43], [60]. Fullmer and colleagues demonstrated via terminal restriction fragment length polymorphism that the subgingival microbial profiles differed significantly between active smokers and at 6 and 12 months following smoking cessation [43]. Moreover, since microbes residing in a biofilm state are known to have increased resistance to antibiotics, tobacco induced biofilm formation may contribute to the refractory nature of many respiratory infections found in smokers.
